# Dynamic DNA Origami Devices: from Strand-Displacement Reactions to External-Stimuli Responsive Systems

**DOI:** 10.3390/ijms19072114

**Published:** 2018-07-20

**Authors:** Heini Ijäs, Sami Nummelin, Boxuan Shen, Mauri A. Kostiainen, Veikko Linko

**Affiliations:** 1 Biohybrid Materials, Department of Bioproducts and Biosystems, Aalto University, P.O. Box 16100, 00076 Aalto, Finland; heini.ijas@aalto.fi (H.I.); sami.nummelin@aalto.fi (S.N.); boxuan.shen@aalto.fi (B.S.); mauri.kostiainen@aalto.fi (M.A.K.); 2 Department of Biological and Environmental Science, University of Jyväskylä, P.O. Box 35, 40014 Jyväskylä, Finland; 3 HYBER Center of Excellence, Department of Applied Physics, Aalto University, 00076 Aalto, Finland

**Keywords:** DNA nanotechnology, DNA origami, self-assembly, molecular devices, mechanical movement, robotics

## Abstract

DNA nanotechnology provides an excellent foundation for diverse nanoscale structures that can be used in various bioapplications and materials research. Among all existing DNA assembly techniques, DNA origami proves to be the most robust one for creating custom nanoshapes. Since its invention in 2006, building from the bottom up using DNA advanced drastically, and therefore, more and more complex DNA-based systems became accessible. So far, the vast majority of the demonstrated DNA origami frameworks are static by nature; however, there also exist dynamic DNA origami devices that are increasingly coming into view. In this review, we discuss DNA origami nanostructures that exhibit controlled translational or rotational movement when triggered by predefined DNA sequences, various molecular interactions, and/or external stimuli such as light, pH, temperature, and electromagnetic fields. The rapid evolution of such dynamic DNA origami tools will undoubtedly have a significant impact on molecular-scale precision measurements, targeted drug delivery and diagnostics; however, they can also play a role in the development of optical/plasmonic sensors, nanophotonic devices, and nanorobotics for numerous different tasks.

## 1. Introduction

In his idiosyncratic talk “There’s plenty of room at the bottom” in 1959, Richard Feynman envisioned that it should be possible to build nanoscale machines that could carry out chemical synthesis through mechanical movement [1]. He also presented Albert R. Hibbs’s idea of miniature surgical robots that could perform predefined tasks in the human body [1]. Now, almost 60 years later, thanks to modern biology, we know that the human body is actually a large-scale biofactory that is comprised of a great number of tiny and accurate nanomachines, such as motor proteins and enzymes, as a result of billions of years of evolutionary processes on Earth. However, we are not merely products of those natural nanomachines or simply hosts to them, but we are also able to look at them with our state-of-the-art microscopes, and even more interestingly, to create artificial and completely new nanodevices. In other words, we are putting Feynman’s idea into practice.

At the time of Feynman’s talk, the structure of double-stranded DNA (dsDNA) was resolved just six years before [2]. However, it was known that DNA carries genetic information and how DNA strands hybridize to each other following Watson–Crick base-pairing rules [2]. Nevertheless, it took almost 30 years before the potential of DNA molecules as programmable construction materials [3]—and not merely as the storage of genetic information—was proposed by Nadrian Seeman [4]. Since then, the field of structural DNA nanotechnology constantly grew and it started to truly flourish during the last decade [5,6]. Today, researchers routinely use DNA to build not only static two- and three-dimensional (2D and 3D) nanostructures via self-assembly [4,5,6,7,8,9,10,11], but also dynamic and precise nanodevices and robots [12,13] that Feynman could hardly imagine. As a matter of fact, Feynman’s statement “Biology is not simply writing information; it is doing something about it” [1] is literally realized in the case of DNA molecules and DNA nanotechnology.

Although structural DNA nanotechnology constantly evolved during the last 35 years, starting from Seeman’s vision of using DNA junctions and lattices to build DNA crystals [6,7], the recently witnessed big boom in the field started from the invention of 2D DNA origami in 2006, a technique developed by Paul Rothemund [14]. DNA origami is based on a long single-stranded DNA (ssDNA) scaffold that is folded into a desired nanoscale shape with the help of dozens of short oligonucleotides [14]. Since 2006, the method was extended to 3D shapes [15,16], designs with curvatures and twists [17,18], wireframe-based and automatically designed structures [19,20,21], and assemblies that can reach micrometer or gigadalton scales [22,23]. Inspired by DNA origami, scaffoldless methods that are based on brick-like assemblies were also developed [24,25].

The benefits of using the DNA origami technique are not only the virtues of custom nanoscale shapes, but also extremely accurate molecular-scale positioning and patterning. These features can be used in controlling chemical reactions [26,27,28], creating tunable plasmonic systems [29,30], and building carriers for drug delivery [12,31,32,33,34,35]. Precise and addressable DNA origami can also be used as rulers and in optical super-resolution imaging [36], forming crystals and nanoparticle superlattices [37,38], and creating inorganic nanostructures [39]. Recently, it was also observed that DNA origami structures are more resilient than previously understood [40], and that the mass production of DNA origami is affordable [41]. Therefore, highly versatile and modular DNA origami is about to become a standard molecular-scale tool in numerous laboratories.

**Scheme 1:**
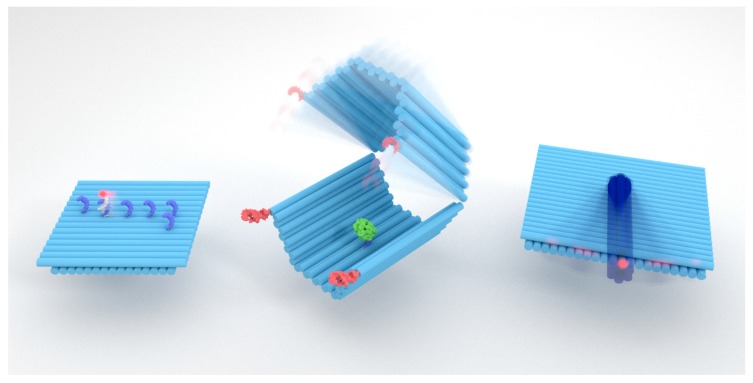
Artistic rendering of selected examples of dynamic DNA origami devices: (**Left**) a cargo-sorting robot walking on a DNA origami-templated track [42]; (**Middle**) a logic-gated DNA origami “nanopill” that selectively displays the loaded cargo [12]; (**Right**) a DNA origami robotic arm that performs rotational movement under an electric field [43].

In this review, we discuss DNA origami nanostructures that can be used as dynamic and controllable nanodevices, such as walking robots [42], logic-gated nanopills [12], and rotors [43] (see Scheme 1). The development of such molecular machines is based on tailoring the DNA sequences in such a way that the structures firstly self-assemble into desired shapes, and are thereby able to perform predefined tasks via translational or rotational movement. In this respect, dynamic DNA origami devices can be considered analogous to protein shapes and functions that are encoded in the sequences of the polypeptide and nucleic-acid molecules [8]. Importantly, DNA origami provides a straightforward route from sequence design to actual shapes, unlike protein synthesis. However, it is noteworthy that de novo protein design allows the synthesis of completely new proteins with tailored functions [44]. A combination of these two techniques would have potential to revolutionize biomedicine and molecular nanotechnology.

In many dynamic systems, the ability to simulate molecular motion and fluctuations becomes increasingly important. There are ways to predict DNA origami dynamics based on rigid-beam models (CanDo) [45,46], atomistic molecular-dynamics simulation [47], and coarse-grained models (oxDNA) [48,49]. Additionally, mass-weighted chemical elastic network models (MWCENM) and symmetry-constrained elastic network models (SCENM) [50] can be used to estimate the structural fluctuations.

Although the focus of this review is on the DNA origami-based devices, it is noteworthy to mention that diverse DNA-based molecular machines were already introduced years before DNA origami. Famous examples include a machine that performs movement based on a DNA conformation change (DNA switches between B- and Z-forms) [51], and DNA tweezers that can be fueled by additional DNA strands to switch the configuration between open and closed states [52]. By taking advantage of simple DNA nanostructures, it is possible to form nanomechanical devices with different rotational or translational states [53,54], and to control their movement using, e.g., RNA strands instead of DNA [55]. Later on, DNA nanostructure-based tweezers [56,57], whose arms can be further equipped with enzymes to facilitate control over chemical reactions were proposed. There are also numerous DNA-based walkers that utilize strand-displacement reactions and employ so-called toehold exchanges. Toeholds are short ssDNA overhangs at the end of dsDNA molecules that firstly bind to reactant ssDNA, i.e., they serve as “docking sites” that initiate the strand-displacement reactions. Such devices were extensively reviewed in References [58,59].

Here, we review dynamic DNA origami devices by dividing the discussion into sections using the criteria of interaction type. In Section 2, we firstly discuss DNA origami assemblies with DNA–DNA interactions, i.e., the mechanical design of DNA origami and the systems that take advantage of strand-displacement reactions, base-stacking interactions, or transient DNA binding. Section 3 is devoted to dynamic DNA origami devices that move due to some other molecular interaction. In other words, the molecular interaction produces a desired movement, or alternatively, the interaction can be characterized using the device as a measurement tool. Section 4 reviews the DNA origami movement due to external stimuli such as light, temperature, pH, and electromagnetic fields, and the devices that can be utilized to probe multiple interactions. Section 5 concludes the discussion and gives future perspectives in this immensely growing field.

## 2. DNA–DNA Interactions: Strand Displacement, Base Stacking and Transient Binding

Here, dynamic DNA origami systems based on DNA–DNA interactions are introduced. This section covers devices whose movements rely on base-pairing (strand-displacement) systems that exhibit dynamic behavior based on DNA base-stacking interactions and tight-fitting DNA components.

**Figure 1:**
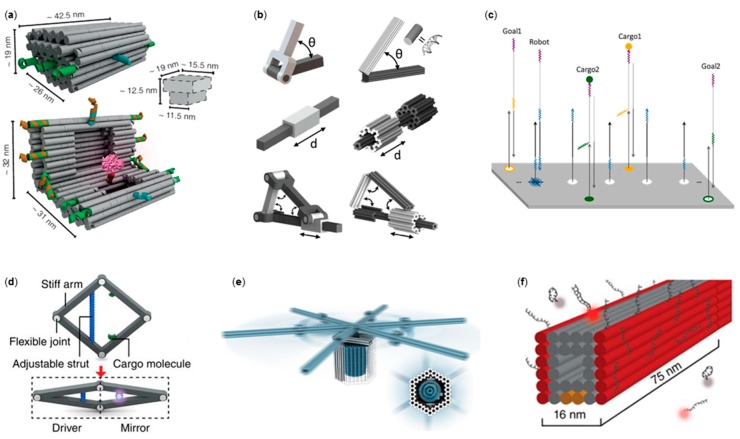
DNA origami mechanics via DNA–DNA interaction. (**a**) A DNA nanovault that displays cargo when opened via strand displacement [61]; (**b**) DNA origami nanomechanics [67]; (**c**) A robot that picks up cargo and delivers it to a goal on top of a DNA origami [42]; (**d**) A DNA origami actuator; movement on the left (driver) side is mirrored to the right side [70] (**e**) A DNA origami rotary apparatus constructed from tight-fitting components [78]; (**f**) Super-resolution imaging with DNA origami by taking advantage of transient DNA binding [81] (**a**) is reproduced with permission from the authors of [61], published by Nature Publishing Group, 2017; (**b**) is reproduced with permission from the authors of [67], copyright National Academy of Sciences 2015; (**c**) is reproduced with permission from the authors of [42], copyright The American Association for the Advancement of Science, 2017; (**d**) is reproduced with permission from the authors of [70], published by Nature Publishing Group, 2016; (**e**) is reproduced with permission from the authors of [78], published by The American Association for the Advancement of Science, 2016; (**f**) is reproduced with permission from the authors of [81], copyright Nature Publishing Group, 2014.

As early as 2009, Andersen et al. [15] fabricated a hollow DNA origami “cuboid” from six 2D origami sheets. The 3D box contained a controllable lid functionalized with a “lock–key system” comprised of dsDNAs with sticky-end extensions. Fluorescent dyes, Cy3 and Cy5, embedded in the opposite faces of the box, facilitated the detection of irreversible lid-opening through a strand-displacement reaction using Förster resonance energy transfer (FRET). Three years later, Zadegan et al. [60] demonstrated reversible opening and closing of a lid in a hollow 3D DNA origami box as a response to supplied opening and closing ssDNA keys. In 2017, Grossi et al. [61] constructed a DNA nanovault with a similar reversible opening/closing mechanism (Figure 1a). They were able to encapsulate a single enzyme inside the vault and demonstrate that closing the nanovault resulted in a notable reduction in enzyme activity. Another type of a dynamic DNA origami device was introduced by Tomaru et al. [62], who created a DNA origami comprised of a “rotor” and a “base” component, connected with a short scaffold segment. They demonstrated a step-wise rotation of a rotor on top of the base through sequential strand-displacement reactions.

Recently, Selnihhin et al. [63] applied a toehold-mediated strand-displacement-based lock–key system in a dynamic DNA origami beacon designed for high-sensitivity biosensing. By functionalizing the device with high numbers of fluorophores interacting via FRET in a closed-state device, they could demonstrate that opening the devices with target DNA keys caused a detectable decrease in FRET efficiency, even at DNA concentrations as low as 100 pM.

To build dynamic devices, it is essential to understand the mechanical behavior of DNA origami and their responses under external physical forces. Numerous studies exploited these aspects of DNA nanostructures [64]; for example, Zhou et al. [65] designed and characterized a tunable DNA origami structure with a compliant part able to bend into different angles under the tension caused by ssDNAs with various lengths. Later, they also studied a four-bar bistable mechanical system with a designed energy landscape, and showed that the conformational dynamics of the device could be controlled via strand displacement [66]. In 2015, Marras et al. [67] demonstrated mechanical designs of DNA origami inspired by macroscopic devices, including a hinge (rotational motion), a slider joint (translational motion), and a complex crank–slider mechanism integrated from the former two (see Figure 1b). In addition, a Bennett linkage which could be actuated via strand displacement was also characterized.

As another class of DNA–DNA interaction, a number of non-autonomous, autonomous, and directed DNA walkers were introduced and analyzed both experimentally and theoretically [58,59,68]. By taking advantage of the DNA origami addressability and programmability, the environment where the walker or robot is moving can be defined and precisely tuned. One example of such a system was presented by Lund et al. [69], where “molecular spiders” made of a streptavidin body equipped with three catalytic deoxyribozyme legs were set to autonomously move along the predefined path on top of a DNA origami template. A very recent and sophisticated example of DNA-assembled robotics on a DNA origami platform was created by Thubagere et al. [42]. They developed an algorithm for sorting two types of cargo and their destinations on a DNA origami platform (Figure 1c). The DNA robot constructed from three functional domains was able to pick up the cargo and release it at the desired location. The movement of the robot was solely based on random walk, and thus, it did not require any additional energy to operate. The robot performed on average 300 steps during the cargo sorting, which is a huge improvement (one to two magnitudes) on previously reported DNA walkers that performed tasks while walking.

In 2016, Ke et al. [70] presented a nanoactuator design consisting of four DNA origami beams linked into a rhombus shape via flexible ssDNA joints (Figure 1d). The opening angle of the device was controlled through ssDNA lock strands of different lengths. Attaching two halves of enhanced GFP (eGFP) to the device and closing the device with short locking strands was shown to bring the halves together and restore fluorescence of the protein. A similar working principle was amplified into a large-scale reconfiguration of a 2D origami lattice by Choi et al. [71]. They built a DNA accordion rack from long DNA beams connected via multiple flexible joints. The aspect ratio of the whole lattice could be controlled by adding DNA lock strands at selected positions in the structure. By applying toehold-mediated strand-displacement processes, multiple rounds of conformational switching could be demonstrated. In addition to using strand displacement, the needed DNA trigger can also be grown using DNA polymerase, as described by Agarwal et al. [72]. They demonstrated the straightening and rigidifying of a deformed wireframe DNA origami having ssDNA gaps by growing a complementary gap-filling strand on site by DNA polymerase.

Base stacking of blunt-ended dsDNA segments can form strong attractive interactions between different DNA nanostructures, or within a single device [73,74]. Gerling et al. [74] showed that transitions between different conformational states in various DNA origami designs could be controlled by adjusting the strength of base-stacking interactions between shape-complementary parts with cation concentration or temperature. A tweezer-type structure based on the design from Reference [74], and its rapid dynamics triggered by cation concentration change were recently characterized using high-speed atomic force microscopy (AFM) [75] and small-angle X-ray scattering (SAXS) [76]. Base-stacking interactions were also used as a driving force in conveying information in large 2D DNA origami arrays [77]. The system constructed by Song et al. [77] consisted of multiple interconnected trapezoidal “antijunction units”. Selected units were firstly locked into a defined conformation through the addition of trigger strands complementary to ssDNA regions at the edges of the units. Neighboring units would then switch into the same conformation, since new base-stacking interactions formed in the process would lower the energy of the system. This was seen to generate a cascade of conformational switching, so that all the units in the system would eventually be found in the same conformational state.

DNA–DNA interactions, including base stacking, can also be used to assemble complex nanomachines from multiple DNA origami elements, as demonstrated by Ketterer et al. [78]. They manufactured a miniature rotary apparatus analogous to F1F0-adenosine triphosphate (ATP) synthase (Figure 1e). The device was constructed from multiple tight-fitting DNA origami components by guiding the self-assembly with specific base-stacking interactions or DNA hybridization events. Based on single-particle fluorescence microscopy recordings, the devices were shown to exhibit random Brownian rotary motion. Controlling the movement of such devices with external triggers could lead to the realization of intricate DNA-based nanomachines.

Recently, dynamic DNA devices were combined with plasmonic systems, whose optical responses are extremely sensitive to the relative positions and orientations of the components. Kuzyk et al. [79] assembled a metamolecule from two gold nanorods (AuNRs) and two interconnected DNA origami beams. The origami beams were connected via a single Holliday junction in the middle, and ssDNA at the ends of the beams were used in strand displacement to switch the structure between a closed state and an open state. By switching the origami, the relative angle between the two AuNRs was altered, which resulted in a change in the circular dichroism (CD) signal (see Section 4 for similar systems with other stimuli). In addition to the dynamic DNA origami device, oligonucleotide-functionalized AuNR could also walk on a static origami via strand displacement [80].

Although not being an actual device, it is worth mentioning that the dynamic interaction between short oligonucleotides has promising applications in super-resolution imaging. The so-called transient binding describes a temporary binding of complementary DNA strands at a temperature close to the melting point of oligonucleotides with specific sequences and lengths. DNA origami with docking strands, which allow the transient binding of dye-labeled oligonucleotides, were used in DNA point accumulation for imaging in nanoscale topography (DNA-PAINT) in super-resolution microscopy [36,81,82] (see Figure 1f). Furthermore, super-resolution imaging of whole cells was recently realized by combining DNA-PAINT with spinning-disc confocal microscopy [83].

## 3. DNA Origami Devices with Molecular Interactions

In this section, DNA devices with dynamic properties mediated by molecular interactions (other than DNA–DNA) between the device and other molecules in the solution are discussed. The scale of the dynamic devices ranges from tools designed for measuring or detecting a specific molecular interaction to aptamer-functionalized objects for nanorobotics, computing, and drug delivery.

**Figure 2:**
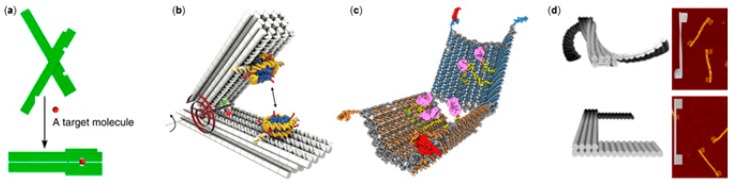
DNA origami devices with molecular interactions. (**a**) DNA origami pliers or forceps that exhibit conformational change upon a target molecule binding [84]; (**b**) A DNA origami measurement device equipped by nucleosomes to probe nucleosome–nucleosome interaction [85]; (**c**) A logic-gated nanorobot that displays cargo when specific antigens bind to aptamer-encoded DNA locks [12]; (**d**) DNA origami twisting and rotation through the application of DNA intercalating molecules [94]; (**a**) is reproduced with permission from the authors of [84], published by Nature Publishing Group, 2011; (**b**) is reproduced with permission from the authors of [85], published by The American Association for the Advancement of Science, 2017; (**c**) is reproduced with permission from the authors of [12], copyright The American Association for the Advancement of Science, 2012; (**d**) is reproduced with permission from the authors of [94], copyright American Chemical Society, 2016.

An early example of dynamic DNA origami devices used in molecular detection is the single-molecule beacons presented by Kuzuya et al. in 2011 [84] (Figure 2a). These “DNA origami pliers” or “DNA origami forceps” consist of two rigid beams connected via a flexible DNA crossover region, which allows the arms to rotate relative to each other. When a target such as protein, metal ion, or human microRNA (miRNA) binds to both arms of the device, the arms are locked into a parallel orientation. Thus, single-molecule binding events are amplified into a major conformational change that can be detected using transmission electron microscopy (TEM) or AFM. 

Other neat examples showing the potential of DNA origami-based measurement tools are the various studies carried out with DNA origami devices and nucleosomes [85,86,87]. Funke et al. [85] and Le et al. [86] both introduced measurement devices with similar hinge-like designs, where two DNA origami arms were joined together at one end via a flexible ssDNA hinge (Figure 2b). The system presented by Funke et al. was initially introduced as a static tool for placing molecules at set distances with extreme precision [88]. In the nucleosome studies, the molecular interaction under interest changed the opening angle of the device, which could then be used as a measure of the interaction strength. The device was used to study both nucleosome unwrapping at different ionic strengths [86] and the strength of attractive interactions between two nucleosomes [85]. Le et al. [87] used their device for probing various properties of nucleosome–DNA interaction, such as nucleosomal end-to-end distance, nucleosome conformation, and nucleosome stability.

The previous examples of molecular measurement devices all share a relatively similar working principle: molecular binding or interaction under interest converts the device into a discrete, relatively immobile orientation, which is then characterized. In contrast to this, Hudoba et al. [89] measured compressive depletion forces in a solution with a dynamic device that constantly fluctuated between an open (uncompressed) and a closed (compressed) state. Increased depletion forces caused by molecular crowding agents, particularly by poly(ethylene-glycol) (PEG), were observed to shift the dynamics of the device more toward the closed state.

DNA origami devices with specific interactions with other biomolecules of interest can be constructed with the help of aptamers. Aptamers are oligonucleotides which bind a specific target molecule with high affinity. One type of dynamic aptamer-functionalized system is a container that is held closed by the aptamer regions hybridized to complementary DNA strands [12,90]. The container is released into an open state when the aptamers come in contact and bind to their target molecule, which creates an intriguing potential to use these types of DNA devices as specifically targeted drug-delivery vehicles.

A famous example of an aptamer-functionalized DNA origami container is a logic-gated nanorobot introduced by Douglas et al. [12] (Figure 2c). The robots were equipped with different combinations of aptamers. The robot held in a closed state would open and release its cargo only when two different triggers were simultaneously encountered, thus creating a logical AND-gate. In a later study, Amir et al. [91] developed the idea of recreating logical functions and performing molecular computing using DNA nanorobots even further. When aptamer-functionalized robots were mixed in defined molar ratios with robots that could either activate or deactivate the original robots via DNA–DNA interactions, the robot mixtures could emulate a variety of logical functions and perform rudimentary computing inside living cockroaches. Recently, it was demonstrated that a mixture of three interacting robots (similar to those in Reference [91]) could behave according to Isaac Asimov’s three laws of robotics [92,93]. With these logic-gated DNA nanorobots and a microRNA molecule (a human miR-16 analog) as a damage signal of the system, the authors were able to recreate Isaac Asimov’s famous “Runaround” scenario (dynamics between robot populations), using approximately 100 billion robots [92].

Recently, the in vivo therapeutic potential of aptamer-functionalized DNA origami nanorobots was shown by Li et al. [90], who designed a tubular DNA origami device by rolling up 2D origami sheet with the help of nucleolin-binding aptamers. The devices were shown to unroll and expose active thrombin in the vicinity of targeted endothelial tumor cells, leading to the inhibition of tumor growth.

Dynamic DNA origami devices are often constructed by linking rigid dsDNA elements with flexible hinges or pivot points formed of ssDNA. Chen et al. [94] studied the possibility of inducing controllable dynamic behavior in structures consisting solely of dsDNA. They utilized DNA-binding adducts, such as ethidium bromide (EtBr), which intercalate between DNA base pairs and cause torsional deformation of dsDNA by unwinding the duplex. By increasing the intercalator concentration in the solution, they demonstrated a significant twist along the length of the whole DNA origami (Figure 2d). The change was shown to be fine-tunable via intercalator concentration, and partially reversible when intercalators were removed via the addition of competing DNA strands.

In addition to moving DNA origami devices, DNA origami itself can act as a template in a highly dynamic assembly. As an intriguing example of such a system, a programmable DNA origami rod was used as a cargo mimic for motor proteins [95]. By controlling the number and types of proteins linked to the cargo mimic, a molecular-scale tug-of-war can be assembled for probing the collective motility of the selected motor proteins. Similarly, dynamic DNA origami diffusion can be assisted by lipid bilayers [96,97,98]. This type of lipid-assisted diffusion is not fully controllable and does not exactly fall into the category of DNA origami devices; however, interestingly, by employing lipid bilayers, DNA origami can be dynamically arranged into well-defined lattices and other higher-order assemblies. The DNA origami diffusion on top of a lipid layer can be tuned using cholesterol modifications in DNA origami, taking advantage of lipid membrane phases (liquid-disordered or solid-ordered) or by adjusting cation concentration. Therefore, it is likely, that a combination of DNA origami with proteins and lipids may find uses in developing dynamic nanomachines.

## 4. DNA Origami Devices Triggered by External Stimuli or Multiple Interactions

This section deals with DNA origami nanosystems in which plasmonic effects, or dynamic and controllable movement, e.g., switching or rotation, are induced using a wide spectrum of external stimuli ranging from photoregulation to pH and thermally directed assembly to electromagnetic fields.

**Figure 3:**
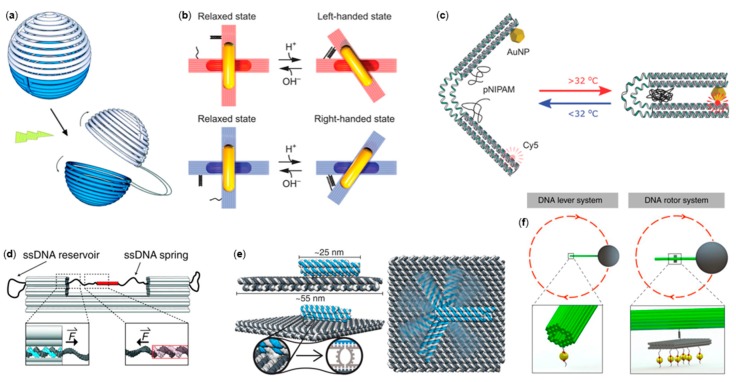
DNA origami movement using stimuli. (**a**) A spherical DNA origami container that can be opened by light [100]; (**b**) Reconfigurable chiral plasmonic metamolecules [103]; (**c**) A thermoresponsive actuator [106]; (**d**) An autonomous nanoscopic force clamp [107]. (**e**) An electric-field-directed robotic arm [43]. (**f**) Magnetic actuators; a lever system and a rotor [111]. (**a**) is reproduced with permission from the authors of [100], copyright The Royal Society of Chemistry, 2015; (**b**) is reproduced with permission from the authors of [103], published by The American Association for the Advancement of Science, 2017; (**c**) is reproduced with permission from the authors of [106], copyright John Wiley and Sons, 2018; (**d**) is reproduced with permission from the authors of [107], copyright The American Association for the Advancement of Science, 2016; (**e**) is reproduced with permission from the authors of [43], copyright The American Association for the Advancement of Science, 2018; (**f**) is reproduced with permission from the authors of [111], published by Nature Publishing Group, 2018.

Yang et al. [99] were among the first to show UV-controllable DNA origami structures. They demonstrated the assembly and disassembly of predesigned multi-orientational patterns constructed from rigid DNA hexagons with photoresponsive azobenzene-modified oligonucleotides inserted either into one, two, or three edges. Later on, Kohman and Han [100] demonstrated light-triggered reconfiguration of a hollow spherical DNA nanostructure. The sphere was obtained from two hemispheres linked together via a DNA scaffold, and the sphere was sealed from the equator using nine crossover strands modified with photolabile *o*-nitrobenzyl moieties. Irradiation at the specific wavelength of 302 nm resulted in almost quantitative and irreversible cleavage of nitrobenzyl groups, showing tethered hemispheres in TEM images (Figure 3a). A similar light-controlled container was presented by Takenaka et al. with capture and release properties of gold nanoparticles [101].

Kuzyk et al. [102] designed an elegant, light-driven 3D plasmonic DNA origami nanostructure, which was based on reversible cis–trans photoisomerization of azobenzene units. They used a similar design to that described in Section 2; however, the design now contained azobenzene units that were assembled to form a chiral template with an adjustable angle. Optical control between locked (ca. 50° angle) and relaxed (ca. 90° angle) conformational states was obtained using ultraviolet (UV) and visible (Vis) light illumination. The insertion of two AuNRs into the bundles resulted in a tunable plasmonic chiroptical response with large amplitude modulation upon light stimuli. Elaborating on the previous design [102], Kuzyk et al. [103] demonstrated that the plasmonic metamolecules could also be reconfigured via pH changes, which triggered the selective control over the chiral locked or relaxed state (Figure 3b). The DNA “lock” was based on a DNA triplex formation through pH-sensitive and sequence-specific parallel Hoogsteen interactions occurring between an ssDNA and a duplex DNA. Tuning of the relative contents of TAT/CGC triplets enabled the programmability and discrimination of chiral quasi-enantiomers over a wide pH range. The programmable pH response was also successfully used in the reconfiguration of hexagonal DNA origami dimer and trimer systems using i-motif and Hoogsteen-type interactions [104].

Jiang et al. [105] introduced chiral plasmonic nanostructures by assembling AuNRs into an L-type configuration using two DNA origami triangles as templates. These two templates were specifically connected using a programmable seam that was able to respond to different stimuli. A clear dynamic and reversible plasmonic effect was detected from circular-dichroism spectra when the system was triggered by light (azobenzene-induced G-quadruplex stretching of the seam) or pH changes (folding/unfolding of cytosine-rich i-motifs). The irreversible cleavage of disulfide bonds of the seam via the reduction of glutathione tripeptide and the cleavage of restriction-enzyme-sensitive DNA sequences was also demonstrated.

Turek and co-workers [106] created thermoresponsive DNA origami tweezers in which the actuation was based on reversible temperature-induced coil-to-globule transition of the thermoresponsive polymer poly(*N*-isopropylacrylamide) (PNIPAM). Above the lower critical solution temperature (LCST) of 32 °C, PNIPAM became hydrophobic, causing the folding of both rigid arms (Figure 3c), which was detected as an increased fluorescence of the gold nanoparticle (AuNP) and Cy5 located at the tip of the tweezer in equivalent positions.

Nickels et al. [107] demonstrated that a folded bracket-shape DNA origami functioned as a nanoscopic force clamp for probing multiple different interactions. In this nanodevice, the system of interest (red rectangle, Figure 3d) was linked between two immobile attachment points via entropic ssDNA springs that exert a constant force on a piconewton (pN) scale over time. The force can be tuned by controlling ssDNA strand length, i.e., the entropic force can be increased with reducing nucleotide amount. This leaves ssDNA reservoirs on both sides of the clamp (as seen in Figure 3d). Conformational transitions of the system of interest can be monitored via single-molecule FRET. With this device, switching between two different states of a FRET-pair-equipped four-way Holliday junction was resolved. The authors also demonstrated the sensitivity of the system by characterizing protein-induced DNA bending. Very recently, related to abovementioned force spectroscopy, Dutta and co-workers [108] reported a DNA origami tension probe (DOTP) capable of depicting the traction forces generated by living cells. Various DOTP combinations were employed to map the forces applied by human blood platelets during initial adhesion and activation. Traction forces with piconewton (pN) resolution were measured utilizing tension-to-fluorescence transduction upon unfolding the DNA hairpin that was incorporated into the system.

In addition to the examples above, external electromagnetic fields can be used to manipulate DNA origami movement. Recently, Kopperger and co-workers [43] displayed a truly dynamic DNA origami platform for a nanoscale (up to 400 nm) robotic arm controlled by electric fields. The system was composed of a rigid DNA origami plate equipped with a 25-nm-long six-helix-bundle (6HB) arm attached via ssDNA scaffold crossovers (see the close up in Figure 3e). The flexible joint allowed stochasting switching of the arm due to transient binding, which was detected from FRET signals generated by the donor fluorophore on the tip of the arm and two acceptor dyes mounted on the rectangular base plate. Electrically controlled movement of the robot arm (angular movement up to 25 Hz) was measured when the system was mounted at the center of a cross-shaped electrophoretic chamber with two perpendicular fluid channels. The electric field was applied by two pairs of electrodes inserted in the reservoirs at the ends of the channels. Furthermore, the ability to move inorganic nanoparticles, e.g., AuNRs, using the robotic arm was demonstrated. DNA origami polarizability can also be used to direct and trap DNA origami via dielectrophoresis in a non-uniform electric field, as shown by Kuzyk et al. [109] and Shen et al. [110].

Lauback et al. [111] introduced yet another way of controlling DNA origami movement by employing external magnetic fields. Three quasi-analogous nanostructures, i.e., lever, rotor, and hinge systems (Figure 3f), having diverse angular movement paths, were demonstrated. All constructs were assembled from three components: a base platform, a stiff 56-helix-bundle rotor arm equipped with a micromagnetic bead on the free rotating end, and a ductile pivot anchoring the rotor to the base platform via biotin–streptavidin affinity. The concept allowed sustained rotational motion (up to 2 Hz), the capability of operating up to 80 pNnm of torque, and a definite control (±8° resolution) over the angular conformation.

## 5. Conclusions and Perspectives

“What are the possibilities of small but movable machines? They may or may not be useful, but they surely would be fun to make,” pondered Feynman in his talk [1]. Here, we reviewed DNA origami-based nanomachines that exhibit translational and rotational motion when triggered by various types of stimuli. We also addressed Feynman’s question by discussing the usefulness of these nanomachines. The summary of stimuli/interactions, implementations, and possible applications reviewed in this article are listed in Table 1.

Although the future of artificial DNA-based nanodevices seems bright, there are several challenges and problems that should be resolved. One obvious issue in Feynman’s surgical nanorobot vision is that the DNA structures might degrade in many biologically relevant conditions [40]. Therefore, plenty of strategies for coating and protecting DNA origami were recently introduced [31,112,113,114,115,116]. However, the challenge is to ensure the functionality of the dynamic devices with such protection systems. Even in the simple static systems, functionality may be suppressed due to the protective coating. For example, an enzyme-loaded DNA origami container [117] coated with synthetic polymers [113] showed significant decrease in enzyme activity compared to a non-coated container. Nevertheless, the field of DNA nanotechnology is already at the state where DNA origami-based dynamic drug-delivery systems are increasingly coming into view [32,33,61,90]. Feynman’s postulate regarding mechanical machines is already proved, since we can build accurate DNA origami devices that allow, for instance, molecular-scale precision measurements that are either challenging or not even achievable using other techniques [13]. These devices facilitate the characterization of DNA stacking forces [118], nucleosome–nucleosome and nucleosome–DNA interactions [85,86,87,88], and, for example, the probing of protein–DNA interactions [107]. Moreover, employing external stimuli such as electric [43] or magnetic fields [111], it is possible to bridge microscale manipulation to nanoscale devices, and thereby control the movement of these nanomachines with short response times. Further engineering of these programmable and dynamic DNA origami nanomachines will lead this research field from proof-of-principle examples to actual utility.

## Figures and Tables

**Table 1:** Summary of the reviewed types of DNA origami motion.

Interaction/Stimulus	Implementation	Application
DNA oligonucleotides	Lock–key systems based on toehold-mediated strand displacement Transient binding	Containers [15,60,61] Biosensing [63] Reconfigurable plasmonics [79] Reconfigurable actuators and lattices [70,71] DNA point accumulation for imaging in nanoscale topography (DNA-PAINT) [81,82,83] Robotic walkers [42] Rotary devices [62]
Entropic elasticity and steric effects	ssDNA as an entropic spring	Nanomechanical devices [65,66,67] Force spectrometers [107]
DNA base stacking	Shape-complementary, blunt-ended dsDNA regions	Large-scale assembly [22,73,74]; reconfigurable devices [74]; information relay [77] Rotary devices [78]
Site-specific binding of target molecules	Incorporation of residues with specific chemical reactivity Modified oligonucleotide aptamers	Measurement devices [70,85,86,87,88,89,107] Drug delivery and nanorobotics [12,90,91,92]
Non-site-specific interactions with other molecules	Mixing DNA origami with crowding agents (e.g., poly(ethylene-glycol)), intercalators, or lipid bilayers	Measurement of molecular crowding [89] Fine-tunable twisting motion [94] Lipid-assisted diffusion [96,97,98]
Light (UV/Vis)	Incorporation of photoresponsive molecules, e.g., azobenzenes	Photo-controllable assembly and disassembly of nanostructures [104] Photo-cleavable and -controlled containers [100,101] Reconfigurable plasmonics [102]
pH changes, Hoogsteen interactions	pH-sensitive DNA regions, e.g., i-motifs, G-quadruplexes, or triplex-forming sequences	Reconfigurable plasmonics [103,105] Assembly and disassembly of nanostructures [104]
Temperature changes	Thermoresponsive polymers	Thermoresponsive actuation [106]
Electric or magnetic fields	Polarizability of DNA in electric fields Magnetic beads linked to origami	Rotary devices, hinges, and levers [43,111]
